# Longitudinal Insights Into Childhood Onset Facioscapulohumeral Dystrophy: A 5-Year Natural History Study

**DOI:** 10.1212/WNL.0000000000210059

**Published:** 2024-12-17

**Authors:** Jildou N. Dijkstra, Helena T.M. Boon, Anne Koekkoek, Rianne J.M. Goselink, Maaike M. Pelsma, Nens Van Alfen, Saskia L.S. Houwen-van Opstal, Baziel G.M. Van Engelen, Nicol C. Voermans, Corrie E. Erasmus

**Affiliations:** From the Department of Neurology (J.N.D., H.T.M.B., N.V.A., B.G.M.V.E., N.C.V.); Department of Pediatric Neurology (J.N.D., H.T.M.B., A.K., C.E.E.), Donders Institute for Brain, Cognition and Behaviour, Amalia Children's Hospital, Radboud University Medical Centre, Nijmegen, The Netherlands; Department of Neurology (R.J.M.G.), Jönköping, and Department of Biomedical and Clinical Sciences, Linköping University, Sweden; Department of Rehabilitation (M.M.P., S.L.S.H.), Donders Institute for Brain, Cognition and Behaviour, Amalia Children's Hospital; and Department of Neurology (N.V.A.), Clinical Neuromuscular Imaging Group, Donders Institute for Brain, Cognition and Behaviour, Radboud University Medical Center, Nijmegen, The Netherlands.

## Abstract

**Background and Objectives:**

Facioscapulohumeral dystrophy (FSHD) is an inherited muscle disorder, with childhood onset in 20% of patients. Understanding the natural history of childhood FSHD and identifying clinical and functional outcome measures are crucial for clinical care and future trials.

**Methods:**

In a prospective nationwide FSHD cohort study (iFocus), 20 childhood-onset patients were assessed at baseline, 2 years, and 5 years. Assessments included manual muscle and functional muscle tests, FSHD clinical score (FSHD-CS), FSHD clinical severity scale (FSHD-CSS), and muscle ultrasonography (MUS).

**Results:**

Eighteen patients (aged 2–17 years at baseline) completed the 5-year follow-up. Disease progression varied, with a mean FSHD-CS increase of 1.6. Despite objective disease progression, most participants (89%) did not perceive change. The most sensitive outcome measures were FSHD-CS (standardized response mean [SRM] 1.07), FSHD-CSS score (SRM 0.92), and MUS findings (SRM 0.68). Baseline characteristics did not predict progression.

**Discussion:**

Disease progression was variable and often remained unnoticed by participants. Quality of life improved, and fatigue levels decreased over 5 years. The relatively slow progression and physiologic growth highlight the need for sensitive end points within a 1–2-year time frame. Future pediatric studies should consider larger international cohorts, assess reachable workspace, and include MUS and FSHD functional composite outcome measure (FSHD-COM).

## Introduction

Facioscapulohumeral dystrophy (FSHD) is a progressive hereditary muscular dystrophy, caused by aberrant DUX4 expression.^1^ FSHD manifests in childhood in approximately 20% of patients.^2^ Early-onset FSHD, defined by facial weakness before age 5 and/or scapular weakness before age 10, is typically severe, with systemic features and more muscle weakness than classic-onset FSHD.^2-5^ However, childhood FSHD can also present with milder phenotypes similar to adult-onset FSHD.^6,7^ Despite advancements in understanding adult FSHD,^8,9^ the natural history of childhood FSHD remains less explored. Previous data offer baseline and 2-year follow-up insights.^6,7,10^

Children continue to develop strength and motor skills, potentially improving functional capacity despite disease progression. Hence, growth influences disease progression, making long-term follow-up essential for assessing pediatric FSHD. Currently, no disease-modifying treatments exist for FSHD.^5^ With emerging potential DUX4-targeting therapies^11^ and ongoing adult trials, understanding childhood FSHD's natural history and identifying sensitive end points are crucial. This 5-year prospective nationwide cohort study (iFocus) follows 20 childhood-onset children into adolescence and adulthood. We thus aim to track disease progression, identify predictors, and determine sensitive outcome measures in a population still undergoing normal growth. The long-term follow-up will provide insights into childhood FSHD, improving clinical care and trial readiness.

## Methods

The prospective iFocus cohort was established in 2016 and consists of 20 children with genetically confirmed FSHD. Multisource recruitment was conducted to identify all diagnosed children in the Netherlands. The protocol and description for the baseline and 2-year follow-up characteristics are previously published.^6,7,10^ In this 5-year follow-up study, we added assessments of impairments and physical disability. The full protocol included manual muscle testing; FSHD clinical score (FSHD-CS); FSHD clinical severity scale (FSHD-CSS); motor function measure (MFM); shoulder dimension of the performance of upper limb module (PUL); 6-minute walk test; imaging using standardized muscle ultrasonography (MUS); cardiac and respiratory screening; and patient-reported questionnaires on pain, fatigue, and quality of life (QoL). Methodologic details are presented in eTable 1.

## Results

### Demographics and Genetic Characteristics

Eighteen patients participated (mean current age 15.1 years; eTable 2), 2 of which were evaluated at home. One participant was untraceable, and another declined participation.

### Clinical Assessments

**Figure 1:**
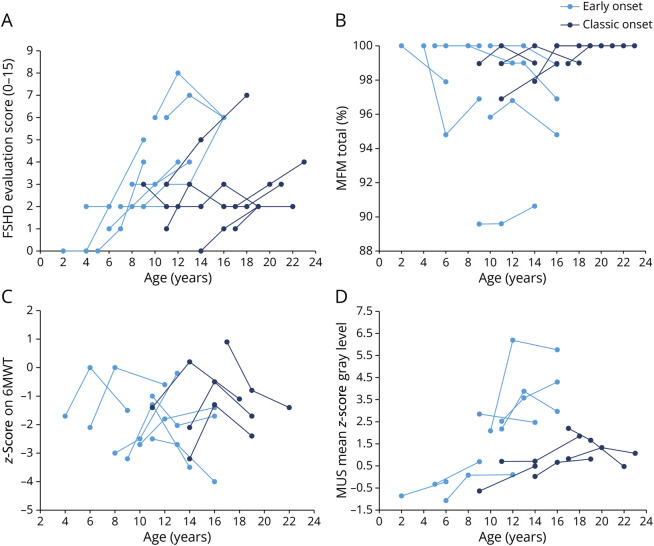
Muscle Function and Imaging in Individual Patients Over 5 Years (A) FSHD clinical score (0–15), (B) performance on motor function measure (0%–100%), (C) performance on the 6-minute walk test (number of standard deviations of mean), and (D) muscle ultrasound mean echogenicity (mean z-score) at baseline, 2-year, and 5-year visits. FSHD = facioscapulohumeral dystrophy.

At 5-year follow-up, most participants (89%) reported stable physical functioning since the previous assessment (at 2-year follow-up). None lost ambulation or required assisted ventilation. The Table summarizes clinical and imaging characteristics including progression rates. The FSHD-CS, FSHD-CSS score, and quantitative MUS findings significantly increased after 5 years. Figure 1 illustrates the variability in disease progression among patients. No significant correlation was found between the number of D4Z4 repeats or age at onset and disease severity (FHSD-CS) (*r* = −0.46, *p* = 0.06, and *r* = −0.37, *p* = 0.13). Similarly, repeat length or age at onset did not correlate with changes in FSHD-CS. Additional results on patient history, clinical assessments, QoL, fatigue, and MUS findings are shown in eTables 3–5.

**Table:** Clinical and Imaging Characteristics at Baseline, 2-Year Follow-Up, and 5-Year Follow-Up

	No. of participants (at baseline-at 2 y-at 5-y follow-up)	At baseline (enrollment)	At 2-y follow-up	At 5-y follow-up	Change from baseline to 5-y follow-up: mean ± SD	*p* of this change
Biometry						
Height (m), mean ± SD	18-16-18	1.42 ± 0.26	1.58 ± 0.18	1.64 ± 0.19	0.21 ± 0.12	<0.001^a^
Weight (kg), mean ± SD	18-16-17	40.7 ± 17.6	51.1 ± 19.2	61.8 ± 26.8	20.1 ± 18.1	<0.001^a^
FSHD-CS (0–15), median (Q1-Q3) (range)	18-18-18	2 (1–3) (0–6)	2.5 (2–3) (0–8)	4 (2–5) (2–7)	1.6 ± 1.5	0.002^b^
FSHD score 0, n (%)		3 (17)	1 (6)	0 (0)		
FSHD scores 1–2, n (%)		9 (50)	8 (44)	7 (39)		
FSHD scores 3–7, n (%)		6 (33)	8 (44)	11 (61)		
FSHD scores 8–15, n (%)		0 (0)	1 (6)	0 (0)		
FSHD-CSS scores (0–10), median (Q1–Q3) (range)	18-18-18	2 (1–2.3) (0–6)	2 (2–3) (0–6)	3 (2–6) (1–6)	1.7 ± 1.9	0.003^b^
Facial weakness^c^, n (%)	18-18-18	14 (78)	17 (95)	18 (100)		0.13^d^
No weakness		4/18 (22.2)	1/18 (5.6)	0/18 (0)		
Moderate weakness		11/18 (50)	13/18 (72.2)	14/18 (77.8)		
Severe weakness		3/18 (16.7)	4/18 (22.2)	4/18 (22.2)		
Scapulohumeral weakness^e^, n (%)	13-14-18	6 (46)	3 (21)	5 (28)		1.0^d^
MRC sum score (0–70), median (Q1-Q3) (range)	18-16-18	70 (67.8–70) (63–70)	68 (66–70) (60–70)	68 (65.8–70) (62–70)	−0.8 ± 1.7	0.06^b^
PUL 1.2 shoulder module score (0–16), median (Q1–Q3) (range)	13-14-18	16 (13.5–16) (8–16)	16 (15.5–16) (1–16)	16 (6.8–16) (0–16)	−3.8 ± 6.9	0.14^b^
6-minute walk test score (z-score) mean ± SD	12-11-16	−2.0 ± 1.6	−1.2 ± 1.0	−1.6 ± 1.1	0.2 ± 1.4	0.69^a^
Motor function measure score (%), median (Q1–Q3) (range)	14-14-17	99 (97.7–100) (89.6–100)	100 (98.4–100) (89.6–100)	99 (97.4–100) (90.6–100)	−0.3 ± 1.5	0.64^b^
Quantitative MUS findings (mean z-score), mean ± SD	13-8-15	1.0 ± 1.5	2.3 ± 2.1	1.6 ± 1.7	0.9 ± 1.3	0.04^a^
Questionnaires						
Pain, n (%)	18-18-18	10 (56)	13 (72)	9 (50)		1.00^d^
Fatigue, n (%)	18-18-18	11 (61)	12 (67)	9 (50)		0.63^d^
NeuroQoL 8-item fatigue bank score, mean SD	9-10-18	1.0	0.9	−0.6		<0.001^a^
Kidscreen total score, mean SD	8-10-17	−0.9	−0.8	0.3		0.01^b^
Kidscreen subdomain scores, mean SD						
Physical well being	8-10-17	−1.4	−2.2	0.2		0.02^b^
Psychological well being	8-10-17	−0.9	−0.4	0.3		0.01^b^
Autonomy	8-10-17	−0.8	−0.5	0.4		0.02^b^
Parent relation and home life	8-10-17	−0.7	0.1	0.5		0.03^b^
Financial resources	8-10-17	−0.7	−0.3	0.3		0.01^b^
Social support and peers	8-10-17	−0.8	−1.0	0.2		0.04^b^
School environment	8-10-15	−0.7	−0.3	0.1		0.03^b^
Social acceptance	6-10-16	−1.5	−1.7	0.1		0.03^b^

Abbreviations: FSHD, facioscapulohumeral dystrophy; FSHD-CS, FSHD clinical score; FSHD-CSS, FSHD clinical severity scale; MRC, Medical Research Council; MUS, muscle ultrasound; PUL, performance of upper limb.

a Paired *t* test.

b Wilcoxon signed-rank test.

c Based on ≥1 point on the facial weakness domain of the FSHD clinical score.

d McNemar test.

e Based on a score of ≤15 on the performance of upper limb shoulder module.

The mean Medical Research Council (MRC) sum score decreased by 0.8 points; the deltoid and iliopsoas muscles were mostly affected, followed by biceps brachii and tibialis anterior muscles. Scapulohumeral weakness, assessed by the PUL 1.2, was present in 5 children (28%), and its prevalence did not increase during follow-up. Both FSHD-CS and FSHD-CSS scores showed significant disease progression, with mean increases of 1.6 and 1.7 points over 5 years, respectively. Despite this overall increase, we observed a wide range of scores, reflecting variability in symptoms (Figure 1). The 6-minute walk test and MFM outcomes did not significantly change.

The mean muscle echogenicity z-score increased significantly by 0.9 in 5 years (*p* = 0.04, Table). The trapezius and rectus femoris were most affected. The rectus abdominis displayed the most substantial and significant increase in z-scores over 5 years (baseline z-score: 1.19; 5-year follow-up z-score: 2.12; *p* = 0.02), followed by the rectus femoris and trapezius muscles. Although there was no correlation between the D4Z4 repeat length and MUS abnormalities (increased echogenicity z-scores) at baseline and follow-up, a strong inverse correlation existed between D4Z4 repeat length and progression of MUS abnormalities over 5 years (*r* = −0.903, *p* < 0.001). The MRC sum score also inversely correlated with quantitative ultrasound abnormalities (r = 0.805, *p* < 0.001). In addition, FSHD-CS correlated moderately with quantitative MUS findings, with higher scores indicating more ultrasound abnormalities (*r* = 0.678, *p* = 0.005). Figure 1D shows changes in mean echogenicity in patients. eFigure 1 provides heatmaps illustrating the severity and changes in MUS abnormalities over time in distinct muscles and the overall distribution of visual graded Heckmatt scores and echogenicity z-scores.

### Responsiveness of Outcome Measures After 2 and 5 Years

**Figure 2:**
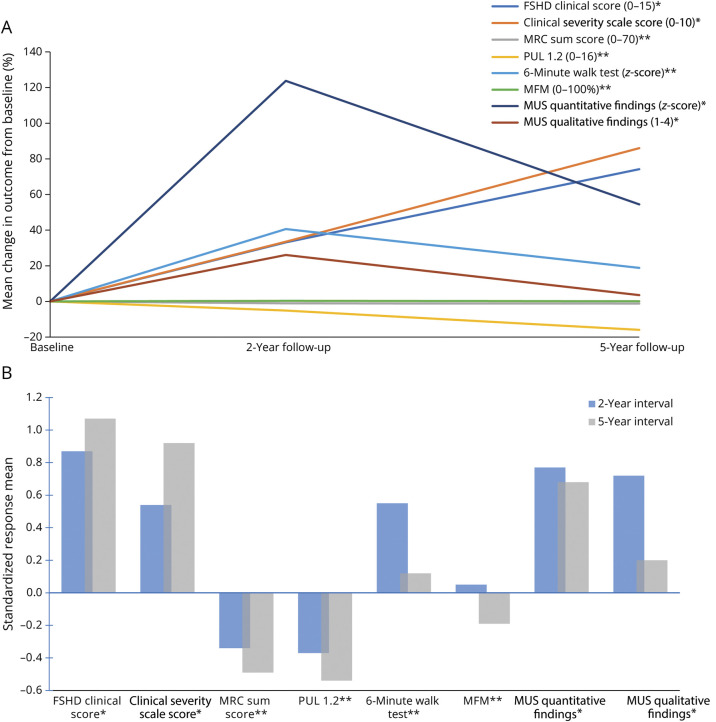
Change in Motor and Imaging End Points Over the 5-Year Follow-Up Period (A) and Responsiveness of Selected End Points Expressed by Standardized Response Mean Values (B) For outcome measures marked with *, a positive result signifies a decline in function. Conversely, for outcome measures marked with **, a positive result signifies an improvement in function. (B) Negative SRMs indicate a reduction in the respective outcome measure whereas positive SRMs signify an increase. An SRM value (whether negative or positive) exceeding 0.8 signifies large responsiveness, a range of 0.5–0.8 moderate responsiveness, and 0.2–0.5 low responsiveness. FSHD = facioscapulohumeral dystrophy; MFM = motor function measure; MRC = Medical Research Council; MUS = muscle ultrasound; PUL = performance of upper limb; SRM = standardized response mean.

The mean percentage change from baseline for various outcome measures over 5 years is shown in Figure 2A, with detailed values in the Table. Figure 2B emphasizes the responsiveness of selected end points using standardized response means (SRMs). After 2 years, FSHD-CS (SRM: 0.87) and quantitative MUS findings (SRM: 0.77) were most responsive. At the 5-year mark, FSHD-CS (SRM: 1.07) and FSHD-CSS scores (SRM: 0.92) exhibited high responsiveness, followed by quantitative MUS findings (SRM: 0.68) and PUL 1.2 shoulder dimension (SRM: −0.54), both moderately responsive. Other end points demonstrated insufficient responsiveness.

### Predictors of Clinical Disease Progression

No baseline parameter correlated with disease progression. Age, sex, and D4Z4 repeat size did not predict the 5-year FSHD-CS progression. There was no association between baseline FSHD-CS, baseline mean echogenicity z-score, or 2-year FSHD-CS change and 5-year progression.

## Discussion

In this prospective nationwide FSHD cohort study (iFocus) of 20 childhood-onset patients, we observed variability of symptoms, severity, and disease progression among individuals. The FSHD-CS was the most sensitive measure of disease progression, followed by the FSHD-CSS and MUS. No predictors of disease progression were identified.

The mean FSHD-CS increased by 1.6 points over 5 years, similar to adult progression, suggesting a comparable pace.^8^ The wide FSHD-CS range in our cohort underscores the variability in symptom progression among children, highlighting the disease's heterogeneous nature. Despite this increase, most children and adolescents did not report worsening conditions. By contrast, we observed improvement in QoL and reduction in fatigue despite disease progression, possibly because of patients developing improved coping mechanisms over time. This aligns with the “disability paradox,” where individuals with severe impairments often report higher QoL scores.^12^ Our findings suggest that, despite disease progression, functional outcomes can improve as children age.

While our study provides valuable insights, several limitations should be considered. The relatively small cohort size and milder phenotype may limit the generalizability of the findings to the broader childhood FSHD population. In addition, the responsiveness of different outcome measures varies across distinct time points and functional tests show a ceiling effect. The ceiling effect in the PUL limits its utility for shoulder function monitoring in our cohort. This highlights the need to evaluate the reachable workspace^13^ as a supplementary measure in children. Moreover, unlike in adults, childhood FSHD exhibits an interplay between growth, enhanced muscle repair,^14^ and disease progression. A more detailed discussion of these limitations, along with further discussion points, is available in eTable 6.

Given slower disease progression, a conventional 1-year trial duration may not be suitable for childhood FSHD without responsive short-term measures. In addition, the variability within individuals and the positive influence of normal motor development complicate end point selection for trials. In response to a lack of robust FSHD specific measurement instruments, the FSHD functional composite outcome measure (FSHD-COM), was published in 2018. We recommend its use in future research. To further enhance trial readiness, we will explore integrating multiple natural history data sets during the upcoming international European NeuroMuscular Centre workshop on childhood-onset FSHD.^16^

To conclude, this study describes the heterogeneous course of childhood FSHD over 5 years. The relatively slow disease progression and the ongoing development of strength in children emphasize the need for identifying sensitive short-term end points. This study contributes to improved counseling for children and their parents and helps to select potential treatment and research goals.

## Appendix Authors

**Table:**

Name	Location	Contribution
Jildou N. Dijkstra, MD	Department of Neurology, Department of Pediatric Neurology, Donders Institute for Brain, Cognition and Behaviour, Amalia Children's Hospital, Radboud University Medical Centre, Nijmegen, The Netherlands	Drafting/revision of the manuscript for content, including medical writing for content; major role in the acquisition of data; study concept or design; analysis or interpretation of data
Helena T.M. Boon, MD	Department of Neurology, Department of Pediatric Neurology, Donders Institute for Brain, Cognition and Behaviour, Amalia Children's Hospital, Radboud University Medical Centre, Nijmegen, The Netherlands	Analysis or interpretation of data
Anne Koekkoek, BSc	Department of Pediatric Neurology, Donders Institute for Brain, Cognition and Behaviour, Amalia Children's Hospital, Radboud University Medical Centre, Nijmegen, The Netherlands	Major role in the acquisition of data
Rianne J.M. Goselink, MD, PhD	Department of Neurology, Region Jönköping County, and Department of Biomedical and Clinical Sciences, Linköping University, Sweden	Drafting/revision of the manuscript for content, including medical writing for content; study concept or design
Maaike M. Pelsma, MSc	Department of Rehabilitation, Donders Institute for Brain, Cognition and Behaviour, Amalia Children's Hospital, Radboud University Medical Center, Nijmegen, The Netherlands	Drafting/revision of the manuscript for content, including medical writing for content; Major role in the acquisition of data
Nens Van Alfen, MD, PhD	Department of Neurology, Clinical Neuromuscular Imaging Group, Donders Institute for Brain, Cognition and Behaviour, Radboud University Medical Center, Nijmegen, The Netherlands	Drafting/revision of the manuscript for content, including medical writing for content; analysis or interpretation of data
Saskia L.S. Houwen-van Opstal, MD, PhD	Department of Rehabilitation, Donders Institute for Brain, Cognition and Behaviour, Amalia Children's Hospital, Radboud University Medical Center, Nijmegen, The Netherlands	Drafting/revision of the manuscript for content, including medical writing for content
Baziel G.M. Van Engelen, MD, PhD	Department of Neurology, Donders Institute for Brain, Cognition and Behaviour, Radboud University Medical Center, Nijmegen, The Netherlands	Drafting/revision of the manuscript for content, including medical writing for content; study concept or design
Nicol C. Voermans, MD, PhD	Department of Neurology, Donders Institute for Brain, Cognition and Behaviour, Radboud University Medical Center, Nijmegen, The Netherlands	Drafting/revision of the manuscript for content, including medical writing for content; study concept or design; analysis or interpretation of data
Corrie E. Erasmus, MD, PhD	Department of Pediatric Neurology, Donders Institute for Brain, Cognition and Behaviour, Amalia Children's Hospital, Radboud University Medical Centre, Nijmegen, The Netherlands	Drafting/revision of the manuscript for content, including medical writing for content; study concept or design; analysis or interpretation of data

## Glossary

FSHD: facioscapulohumeral dystrophy
FSHD-CS: FSHD clinical score
FSHD-CSS: FSHD clinical severity scale
MFM: motor function measure
MRC: Medical Research Council
MUS: muscle ultrasonography
PUL: performance of upper limb module
QoL: quality of life
SRM: standardized response mean
